# Correlation Between Hepatic Waveform Changes on Doppler Ultrasound and Disease Severity in Cirrhotic Patients

**DOI:** 10.7759/cureus.83658

**Published:** 2025-05-07

**Authors:** Sumaiya Latheef, Shivanand Patil, Siddaroodha Sajjan, Pavan Kolekar

**Affiliations:** 1 Radiodiagnosis, Shri BM Patil Medical College Hospital and Research Centre, Bijapur Lingayat District Educational Association (BLDE) (Deemed to be University), Vijayapura, IND

**Keywords:** biphasic waveform, child-pugh classification, cirrhosis, doppler ultrasonography, hepatic vein waveform, liver hemodynamics, monophasic waveform, non-invasive assessment, portal hypertension, triphasic waveform

## Abstract

Background

Liver cirrhosis is the terminal stage of chronic liver disease, marked by progressive fibrosis and vascular remodeling that alters hepatic hemodynamics. Doppler ultrasonography enables non-invasive assessment of these changes by analyzing hepatic vein waveform patterns. This study aimed to evaluate the correlation between hepatic vein waveform alterations and cirrhosis severity, as classified by the Child-Pugh score, and to assess the diagnostic performance of waveform analysis in clinical staging.

Methodology

In this prospective, observational study, 140 patients with clinically or radiologically confirmed cirrhosis underwent Doppler ultrasonography of hepatic veins. Waveform patterns were categorized as triphasic, biphasic, or monophasic and correlated with Child-Pugh classification and other demographic and laboratory parameters. Statistical analyses assessed the strength of these associations and the diagnostic accuracy of waveform classification.

Results

The cohort included 59.3% males and 40.7% females, with 45% aged 41-60 years. According to Child-Pugh classification, 22.9% of patients were Class A, 25% were Class B, and 52.1% were Class C. Hepatic vein waveforms were monophasic in 38.6%, biphasic in 36.4%, and triphasic in 25% of patients. A statistically significant association was observed between waveform patterns and disease severity (p < 0.001), with 70.4% of monophasic and 68.6% of biphasic waveforms seen in Class C patients, and 57.1% of triphasic waveforms in Class A patients. Waveform changes also correlated significantly with age (p = 0.008). Diagnostic evaluation revealed 100% sensitivity and negative predictive value, along with 52.2% specificity and 69.5% positive predictive value for predicting advanced cirrhosis.

Conclusions

Hepatic vein waveform analysis via Doppler ultrasonography is a reliable, non-invasive indicator of cirrhosis severity, demonstrating a strong correlation with the Child-Pugh classification. Its high sensitivity and negative predictive value support its utility as a screening tool for advanced disease. Integrating waveform assessment into routine ultrasound protocols offers valuable prognostic insight without additional cost or complexity.

## Introduction

Cirrhosis remains a significant global health concern and a leading cause of morbidity and mortality worldwide [1]. It is marked by progressive hepatic fibrosis and architectural distortion, which profoundly impact hepatic vascular dynamics [2]. These pathophysiological changes lead to increased intrahepatic resistance, altered portal flow, and eventual hepatic decompensation [3]. In this context, early and accurate assessment of disease severity is essential to guide clinical decision-making, optimize patient outcomes, and allocate healthcare resources efficiently.

Doppler ultrasonography has emerged as a critical non-invasive imaging modality in the evaluation of hepatic hemodynamics [4]. Among its various parameters, hepatic venous waveform analysis has shown promise in reflecting the underlying vascular changes associated with cirrhosis. Normally triphasic in healthy individuals, these waveforms may become biphasic or monophasic as hepatic compliance decreases with disease progression. Such alterations, driven by hepatic congestion, fibrosis, and elevated venous pressures, could potentially serve as indirect markers of cirrhosis severity [5,6].

Despite advances in imaging, the intricate relationship between hepatic venous waveform changes and the clinical severity of liver disease remains underexplored [7]. Chronic liver diseases of varying etiologies, such as viral hepatitis, alcohol-induced liver damage, and non-alcoholic steatohepatitis, share a common endpoint in cirrhosis, where structural and functional liver derangements coalesce [8,9]. Understanding how waveform patterns evolve in response to these pathological processes may enhance our ability to detect and stratify cirrhosis non-invasively, reducing dependence on liver biopsy and enabling timely therapeutic interventions.

Several studies have suggested that changes in hepatic venous waveforms may serve as potential indicators of advanced liver disease [5,7]. However, comprehensive investigations correlating waveform alterations with clinically validated measures of cirrhosis severity remain limited. This study aims to evaluate the correlation between hepatic waveform changes observed on Doppler ultrasound and the clinical severity of cirrhosis.

## Materials and methods

This hospital-based, cross-sectional study was conducted in the Department of Radiodiagnosis at Shri BM Patil Medical College Hospital and Research Centre, BLDE (Deemed to be University), Vijayapura, from May 2023 to December 2024. All participants provided informed written consent, and the study was conducted after obtaining approval from the Institutional Ethics Committee (approval number: BLDE/IEC/940/2023-24). The study followed a structured work plan to ensure the timely execution of each research phase. A total of 140 patients were included in the study. The sample size was calculated based on an anticipated prevalence of monophasic hepatic venous waveforms in 37.5% of cirrhotic patients at a 95% confidence level and an 8% absolute margin of error. The formula used was n = z^2^pq/d^2^, where Z is the desired confidence level, p is the estimated proportion, q is 100 − p, and d is the allowable error.

Patients aged 25 to 75 years with clinical or radiological suspicion of cirrhosis were included in the study. Exclusion criteria comprised the presence of hepatocellular carcinoma, thrombosis of the inferior vena cava, hepatic or portal veins, and congestive heart failure, as these conditions could independently affect hepatic venous flow and confound the study results.

Doppler ultrasonography was performed using two advanced imaging systems, namely, the GE Voluson S8 BT18 and the Versana Premier, equipped with a 3.5 MHz convex transducer. Examinations were conducted following a standardized protocol to ensure reproducibility and accuracy. The right hepatic vein was identified at a distance of 3-5 cm from its confluence with the inferior vena cava, and waveform recordings were taken during quiet respiration. The hepatic vein waveforms were classified into the following three categories: triphasic (showing at least one phase of reversed flow), biphasic (with two forward flow components but no reversal), and monophasic (with a flat, non-pulsatile pattern). This classification enabled a clear and consistent evaluation of waveform changes corresponding to different stages of hepatic dysfunction.

To assess hepatic function and correlate it with Doppler findings, the Child-Pugh scoring system was applied. This included five clinical and laboratory parameters, i.e., serum bilirubin, serum albumin, prothrombin time, presence of ascites, and hepatic encephalopathy. Based on the total score, patients were categorized into Child-Pugh Class A (mild), Class B (moderate), or Class C (severe). All relevant patient data, including demographic details, medical history, ultrasound findings, waveform types, and Child-Pugh classification, were systematically recorded using a structured data collection proforma. Data were entered into Microsoft Excel (Microsoft Corp., Redmond, WA, USA) and analyzed using SPSS version 26 (IBM Corp., Armonk, NY, USA). Frequencies and percentages were calculated for categorical variables, and the chi-square test was employed to assess associations between categorical variables. A p-value <0.05 was considered statistically significant. The results were presented using appropriate tables and graphs for clarity and ease of interpretation.

## Results

**Figure 1:**
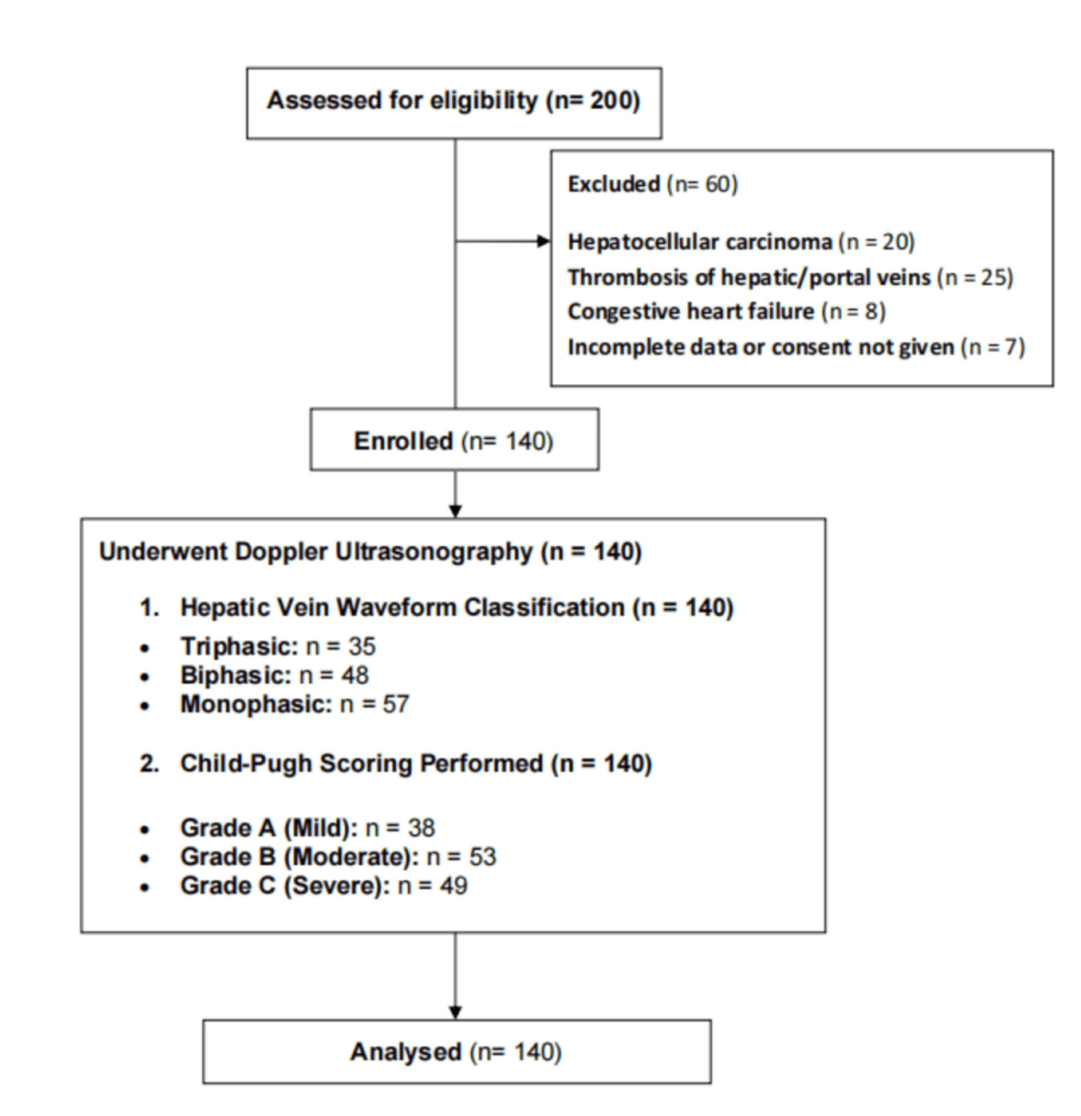
Study flowchart.

The study sample consisted of 140 patients with cirrhosis. Figure 1 presents a flowchart illustrating patient selection. A majority of the participants were in the 41-60-year age range, accounting for 63 (45.0%) patients. Patients aged 20-40 years represented 38 (27.1%), while 39 (27.9%) patients were in the 61-80-year age group. Regarding gender distribution, 83 (59.3%) patients were male, and 57 (40.7%) patients were female. In terms of Child-Pugh classification, the majority of patients were classified as Child-Pugh Class C, comprising 73 (52.1%) patients. The remaining patients were distributed between Class A (32, 22.9%) and Class B (35, 25.0%) (Table 1).

**Table 1:** Patient demographics and Child-Pugh classification. Frequencies and percentages were calculated for categorical variables.

Characteristic	Category	Number	Percentage
Age (years)	20–40	38	27.1%
	41–60	63	45.0%
	61–80	39	27.9%
Gender	Male	83	59.3%
	Female	57	40.7%
Child-Pugh class	A	32	22.9%
	B	35	25.0%
	C	73	52.1%

**Figure 2:**
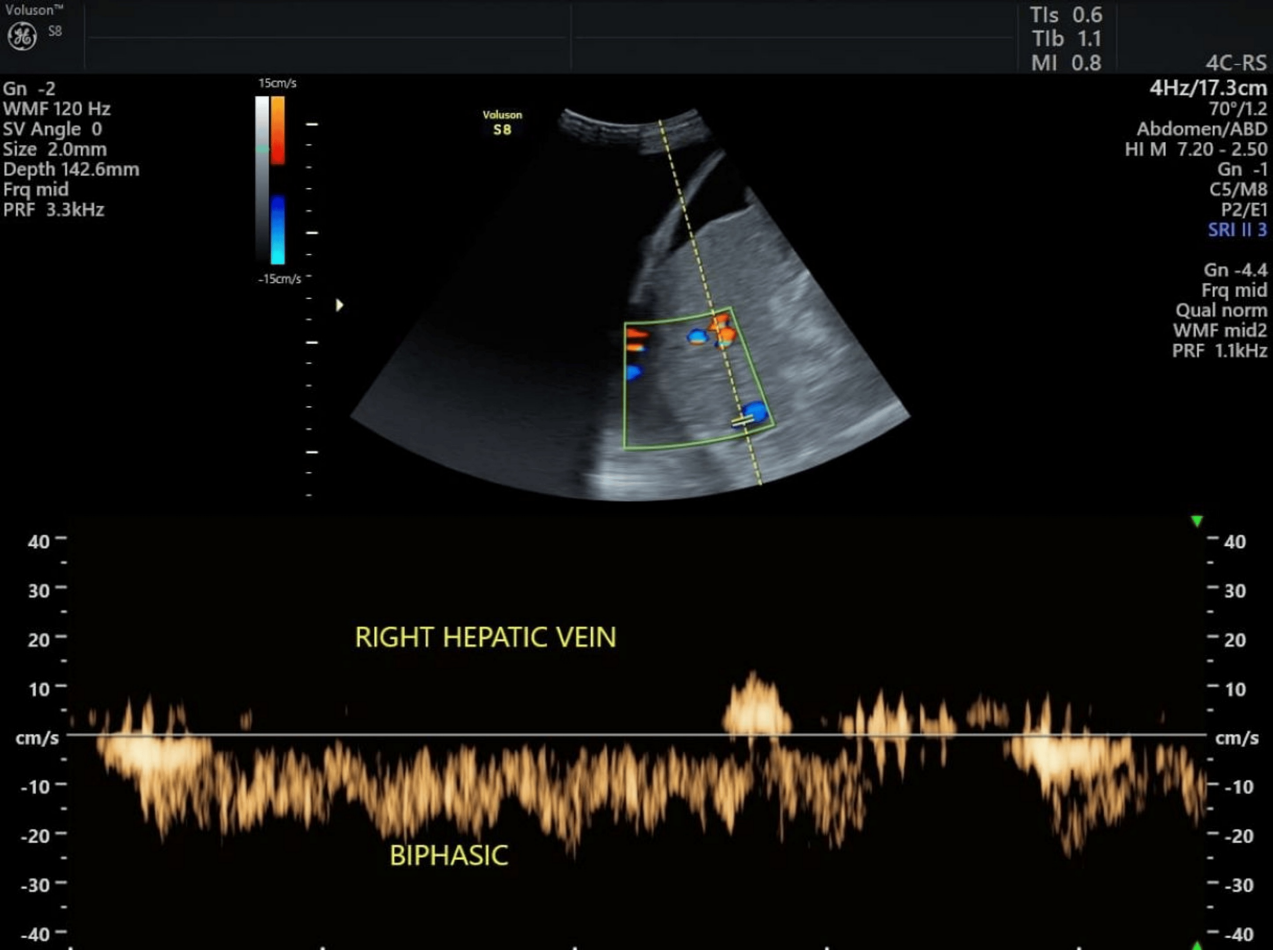
Biphasic waveform in a cirrhotic patient.

**Figure 3:**
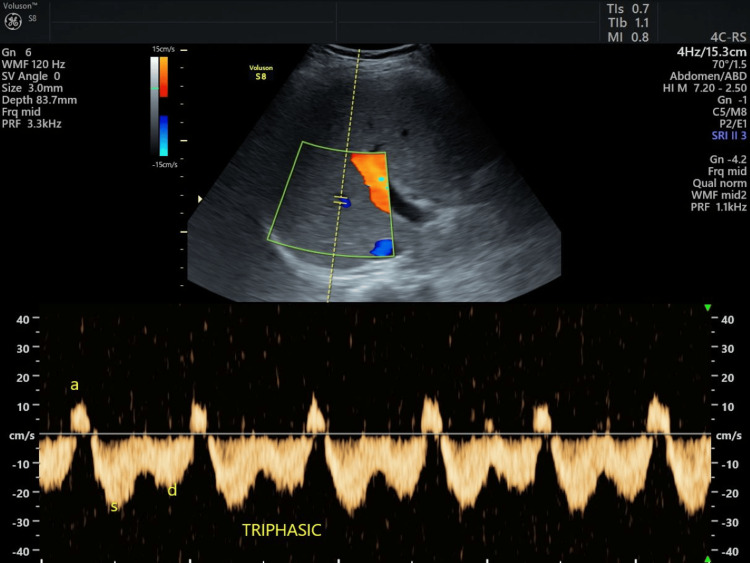
Triphasic waveform in a normal patient.

The distribution of hepatic vein waveform patterns revealed that 54 (38.6%) patients exhibited monophasic waveforms, 51 (36.4%) patients had biphasic waveforms, and 35 (25.0%) patients showed triphasic waveforms. This indicates that more than 70% of the study participants had abnormal hepatic venous flow patterns, with biphasic and monophasic waveforms being the most common (Table 2; Figures 2, 3).

**Table 2:** Hepatic vein waveform pattern distribution. Frequencies and percentages were calculated for categorical variables.

Waveform pattern	Number	Percentage
Triphasic	35	25.0%
Biphasic	51	36.4%
Monophasic	54	38.6%
Total	140	100%

A significant association was observed between hepatic vein waveform patterns and the severity of cirrhosis, as classified by the Child-Pugh system. Among Class A patients, 23 (65.7%) exhibited triphasic waveforms, while 6 (11.8%) had biphasic and 3 (5.6%) had monophasic waveforms. In Class B patients, 12 (34.3%) had triphasic waveforms, 10 (19.6%) had biphasic, and 13 (24.1%) had monophasic waveforms. In contrast, among Class C patients, 38 (70.4%) exhibited monophasic waveforms, 35 (68.6%) showed biphasic waveforms, and none of the Class C patients had triphasic waveforms. The chi-square analysis revealed a significant association between waveform pattern and cirrhosis severity (chi-square = 64.1098, p < 0.0001) (Table 3).

**Table 3:** Association of hepatic vein waveform with Child-Pugh class. Frequencies and percentages were calculated for categorical variables, and the chi-square test was employed to assess associations between categorical variables. A p-value <0.05 was considered statistically significant.

Child-Pugh class	Hepatic vein waveform		
	Biphasic	Monophasic	Triphasic
A	6 (11.8%)	3 (5.6%)	23 (65.7%)
B	10 (19.6%)	13 (24.1%)	12 (34.3%)
C	35 (68.6%)	38 (70.4%)	0 (0%)
Chi-square value	64.1098		
P-value	<0.0001		

The diagnostic performance of hepatic vein waveform patterns assessed by Doppler ultrasound showed perfect sensitivity (100%), with a specificity of 52.2%. The positive predictive value was 69.5%, and the negative predictive value (NPV) was 100%. These results suggest that while Doppler ultrasound is highly sensitive for detecting cirrhosis, its specificity is moderate, indicating its potential utility as an effective screening tool for early-stage disease detection (Table 4).

**Table 4:** Sensitivity analysis of hepatic vein waveform by Doppler ultrasound.

Diagnostic parameter	Value
Sensitivity	100%
Specificity	52.2%
Positive predictive value	69.5%
Negative predictive value	100%

## Discussion

Our study demonstrates a significant correlation between hepatic vein waveform patterns and Child-Pugh classification in cirrhotic patients, providing strong evidence for the role of Doppler ultrasonography as a non-invasive tool for assessing cirrhosis severity. The progressive dampening of the normal triphasic waveform to biphasic and ultimately monophasic patterns reflects the hemodynamic changes associated with advancing cirrhosis. This finding is in line with previous research by Baik et al., who reported that 85% of Child-Pugh Class C patients exhibited monophasic waveforms, while 73% of Class A patients maintained triphasic patterns [10]. This progression in waveform patterns mirrors the increasing vascular resistance and hepatic stiffness observed with cirrhosis, which impairs the transmission of cardiac and respiratory pulsations to the hepatic veins [11]. Intrahepatic portosystemic shunts, which develop as cirrhosis progresses, further alter the pressure gradients and compliance characteristics of the hepatic veins, contributing to these waveform abnormalities.

The diagnostic performance of hepatic vein waveform assessment in our study was exceptional, with sensitivity and NPV both reaching 100%. This suggests that Doppler ultrasound can effectively identify cirrhotic patients with advanced disease, making it a valuable screening tool. Our findings are consistent with those of Chen et al., who reported a sensitivity of 97% and an NPV of 95% for abnormal hepatic vein waveforms in predicting advanced cirrhosis [12]. However, the moderate specificity (52.2%) observed in our study is in line with Mahmoud et al., who found a specificity of 58% for abnormal waveforms in predicting cirrhosis severity [13]. This moderate specificity could be due to extrahepatic factors, such as cardiac function, respiratory patterns, and systemic vascular compliance, which may influence hepatic vein waveforms and lead to abnormal readings even in patients without advanced liver disease.

When comparing hepatic vein waveform analysis with other non-invasive methods for assessing liver disease, such as transient elastography, our study aligns with those of Castéra et al., who reported a correlation coefficient of 0.73 between liver stiffness and Child-Pugh score using elastography [14]. This is slightly higher than the correlation coefficient typically observed for hepatic vein waveforms (r = 0.65-0.70). While elastography is a widely used tool, it has limitations, including technical failures in patients with ascites and obesity, issues that are less commonly encountered with Doppler ultrasound. This highlights the potential advantages of hepatic vein waveform assessment, particularly in settings where transient elastography may not be feasible.

The clinical implications of our findings are significant. The strong correlation between hepatic vein waveform patterns and Child-Pugh classification validates the use of Doppler ultrasound as a reliable non-invasive tool for assessing cirrhosis severity, particularly in clinical settings where comprehensive laboratory testing may be unavailable or delayed. As suggested by Joseph et al., serial assessment of hepatic vein waveforms could serve as a prognostic indicator, with a shift from triphasic to monophasic patterns potentially signalling clinical deterioration before laboratory parameters show significant changes [15]. Additionally, the integration of hepatic vein waveform assessment into a multiparametric ultrasound approach, as proposed by Mahmoud et al., enhances the diagnostic accuracy of non-invasive liver disease assessment [13]. Combining waveform patterns with other ultrasound parameters, such as liver stiffness and portal vein velocity, creates a comprehensive sonographic profile that closely mirrors the clinical and pathological severity of cirrhosis. This approach can offer a practical, cost-effective solution for risk stratification and management optimization in cirrhotic patients.

Despite its promising diagnostic utility, our study has several limitations. The cross-sectional nature of the study prevents us from assessing the temporal progression of waveform changes or their direct correlation with long-term clinical outcomes. Longitudinal studies would provide valuable insights into how hepatic vein waveforms evolve as the disease progresses. Additionally, our study did not stratify patients based on the etiology of cirrhosis, which may influence vascular changes differently across conditions such as viral hepatitis, alcohol-related liver disease, and non-alcoholic fatty liver disease.

## Conclusions

Hepatic vein waveform assessment by Doppler ultrasound emerges as a reliable, non-invasive tool for evaluating the severity of cirrhosis, offering a strong correlation with the Child-Pugh classification. The progressive transition from triphasic to monophasic waveforms mirrors the clinical deterioration seen in cirrhotic patients, reflecting underlying hemodynamic changes that accompany disease progression. Its high sensitivity and NPV make it an invaluable screening method, particularly for ruling out advanced cirrhosis. Although not directly linked to individual laboratory parameters such as albumin or bilirubin, hepatic vein waveform patterns provide significant prognostic value when considered alongside the comprehensive Child-Pugh score. Incorporating this assessment into routine clinical practice can improve patient risk stratification and management, enhancing outcomes without added cost or procedural complexity.
